# Prospective associations between pulse pressure and cognitive performance in Chinese middle-aged and older population across a 5-year study period

**DOI:** 10.1186/s13195-018-0355-1

**Published:** 2018-03-12

**Authors:** Tingting Sha, Wenwei Cheng, Yan Yan

**Affiliations:** Department of Epidemiology and Medical Statistics, Xiangya School of Public Health, Central South University, Xiangya Road 110, Changsha, Hunan 410078 China

**Keywords:** Cognition, Pulse pressure, Aging, Arterial stiffness, Latent growth model

## Abstract

**Background:**

Substantial evidence indicates that the relationship between blood pressure (BP) measures and cognitive functioning is inconsistent, complex, and age-related. Pulse pressure (PP), which can not only reflect arterial stiffness and but also represent the chronic effects of hypertension other than BP itself, has been considered as a better predictor of cognitive impairment. However, evidence on the association of cognitive function with PP has not been investigated extensively. We examined this relationship in a longitudinal study based on the latent growth model (LGM).

**Methods:**

This study was based on a nationally representative sample of Chinese middle-aged and older participants from the China Health and Retirement Longitudinal Study (CHARLS), a prospective observational study conducted from 2011 to 2016. Cognitive performance was assessed on the basis of three measures of cognition. The PP was calculated as the difference of the average values of three systolic and diastolic BP readings. A series of potential confounders were collected in this research. The LGM was used to examine the effects of PP on cognitive performance at three time points. To test the independent effects of PP on the initial level and the subsequent development of cognition, unconditional and conditional models were compared sequentially.

**Results:**

After excluding respondents with missing key variables, we ultimately included 9750 participants in the analysis. Cognitive performance scores and PP showed significant differences across time. After adjustment for the confounders, the standardized coefficients of PP in the LGM indicated negative effects on cognitive performance in elderly Chinese participants at wave 2 and wave 3 (*P* < 0.01). The initial level of PP in the unconditional model was negatively associated with the initial level (β = − 0.25) and the slope (β = − 0.16) of cognition, whereas these effects were attenuated and the association between intercept of PP and slope of cognition became nonsignificant after controlling for the confounders.

**Conclusions:**

The implications of these results demonstrate that a higher PP lowers the cognitive performance of middle-aged and elderly persons independent of a comprehensive set of covariates, but it is not a contributor to the rate of change in cognition.

**Electronic supplementary material:**

The online version of this article (10.1186/s13195-018-0355-1) contains supplementary material, which is available to authorized users.

## Background

According to the World Health Organization, the population aged 60 years and older worldwide is expected to reach more than 2 billion, and the population aged 80 years or older will be quadrupled to 395 million by 2050 [1]. With this rapid global expansion of the aging population, the prevalence of age-related diseases is increasing quickly [2, 3]. Cognitive dysfunction, such as dementia and cognitive impairment, is one of the most challenging diseases and is a leading cause of disability and physical limitations, resulting in a heavy psychosocial and economic burden on both families and society as a whole [4]. China, one of the most rapidly aging societies in Asia [5], has 7.4 million elderly people living with dementia, and the prevalence of dementia is expected to increase to about 18 million by 2030 if no preventive measures are adopted [6].

Substantial evidence indicates that the relationship between blood pressure (BP) measures and cognitive functioning is inconsistent, complex, and age-related. Studies have repeatedly shown that midlife vascular risk factors, such as elevated BP, are regarded as strong, consistent risk factors for age-related dementia or cognitive decline in late life [7–10], but the association between late-life BP and late-life cognition remains contradictory [7, 11–14]. Although systolic blood pressure (SBP) increases with advancing age, diastolic blood pressure (DBP) decreases typically, which causes elevated pulse pressure (PP) [15]. Moreover, PP combines information about SBP and DBP (calculated as the difference between them), which can not only reflect arterial stiffness partly but also potentially represent the chronic effects of hypertension other than BP itself; is more important than traditional BP measures in age-related cognitive decline; and is considered as a better predictor of cognitive impairment than BP [16–20].

Although accumulated evidence shows that PP has a steep age-related increase and is associated with a series of cardiovascular events in older adults [16, 21, 22], evidence on the association of cognitive function with PP has not been investigated extensively. McFall et al. suggested that PP is associated with poor memory tests in elderly adults [17]. Qiu et al. [23] and Wang et al. [24] reported that when they took confounders into account, they found a U-shaped relationship between PP levels and cognitive decline in stroke patients. In contrast, after adjustment for related covariates, other researchers suggested that the correlations between PP and episodic memory were no longer significant [25, 26].

The latent growth model (LGM) is an advanced analytical method that can create random intercepts and slopes to depict the different trajectories over time for each case in a sample [27]. With this model, within-subject variations are allowed at the first level (owing to intraindividual change across time), whereas between-subject variations are estimated at the second level (owing to interindividual differences) [28]. Different from traditional regression models, LGM not only can model intraindividual and interindividual changes by using latent variables but also permits exploration of the antecedents and consequences of change [29].

Because the relationship between PP and cognitive performance is still to some extent inconsistent, we examined this relationship using the LGM in a 5-year follow-up of a nationally representative middle-aged and older Chinese population. The use of the LGM was specified to address the following three questions: (1) Is the initial level of PP associated with cognitive performance among the middle-aged and older Chinese population? (2) Can PP elevation with advancing age magnify the influence of cognitive change? (3) How are these relationships influenced by cardiovascular risk factors such as hypertension, diabetes, and smoking? Understanding the effect of PP on age-related cognitive decline may shed light on preventive strategies.

## Methods

### Data and sample

This study was based on nationwide data derived from the China Health and Retirement Longitudinal Study (CHARLS), a population-based survey conducted by the National School of Development of Peking University, which aimed to provide a high-quality public database with a wide range of information to facilitate the needs of scientific and policy researchers on aging-related issues. A multistage probability sampling design and a probability proportional sampling technique were adopted in the baseline survey to ensure a representative sample. Details of the sampling procedure and a description of the CHARLS are available elsewhere [30]. Briefly, the three-wave surveys of residents aged 45 years or older and their spouses living in China (including 28 provinces, municipal cities, and autonomous regions) were first conducted through face-to-face computer-assisted personal interviewing in June 2011, and these participants were followed every 2 years. The national baseline survey was conducted with 17,708 individual participants (with a response rate of 80.5% at the household level) in 2011–2012. Among the study participants, 13,978 individuals (78.9%) provided anthropometric and physical performance measures. In this group, blood samples were collected from 11,847 individuals, a response rate of 67%. In the second wave, 15,788 of these individuals were successfully reinterviewed in 2013–2014, with follow-up in 2015–2016 (*n* = 15,331). After excluding the missing anthropometric data and blood samples at baseline (*n* = 5906) and the samples with missing data of cognitive performance and BP (*n* = 2052), a subset of 9750 participants were included in the final analysis, with age ranging from 45 to 98 years. The final dataset can be aqcuired in the Additional file 1.

### Measurement

#### Assessment of cognitive performance

Similarly to the cognitive measurements used in the American Health and Retirement Study, we relied on three composite measures of cognitive functioning in this study:

1. *Telephone Interview for Cognitive Status (TICS)*: TICS reflects the mental status of cognition and involves ten questions, including recalling today’s date (month, day, year), the day of the week and season of the year, and serial 7 subtraction from 100 (up to five times). This dimension score is calculated on the number of correct answers, ranging from 0 to 10.
2. *Word recall*: The second measure of cognition relies on word recall of ten words, mainly testing episodic memory of cognition. After the interviewer reads a list of ten Chinese words, the participant is asked to repeat the words in any order immediately. About 4 minutes later, the respondent is asked to recall the list of words again. The word recall score is based on the average of the number of correct answers, ranging from 0 to 10.
3. *Drawing a figure successfully*: The third cognitive measure is a test of the ability to draw a picture of two overlapping pentagons. Respondents who successfully reduce the picture receive a score of 1, and those who fail to do so receive a score of 0. This is an overall measure of the respondent’s cognitive function.

We used the sum of all three of the above measures to represent the respondent’s cognitive status as a whole, with scores ranging from 0 to 21.

#### Assessment of pulse pressure

Resting SBP and DBP were measured by trained nurses at the left brachial artery with the participant in a sitting position. Then BP was remeasured three times with a 45-second interval between each investigated wave. The average of the three SBP and DBP values was obtained to represent the BP estimates. The PP was calculated as SBP − DBP.

#### Assessment of covariates

Analysis of previous studies showed that potential confounders at baseline requiring adjustment were sociodemographic factors, lifestyle and health behaviors, doctors’ diagnoses of chronic diseases, and a series of blood indices. The depressive symptoms of the respondents were evaluated using the ten-item Center for Epidemiologic Studies Depression Scale short form. This scale has been viewed as a valid and reliable instrument for assessment of depression in China [31]. Each item was scored on a 4-point Likert scale, with a total possible score of 30. Activities of daily living (ADL) were assessed using five types of instrumental activities of daily living (IADL) and six types of ADL, including bathing, dressing, eating, indoor transferring, toileting, and continence, with answers varying from “no difficulty” to “much difficulty” and scores ranging from 0 to 3. The sociodemographic factors included age, weight, height, sex, hukou status (Hukou is the registration system in China created in 1955 to restrict internal population movement, especially rural-to-urban migration, divided Chinese into two categories: agricultural hukou (rural hukou) and non‐agricultural hukou (urban hukou). The hukou status is according to parental hukou status, irrespective of child’s birthplace.), marital status, health status before age 16, and education level. The lifestyle behaviors included smoking and alcohol use. Doctors’ diagnoses of chronic diseases reported were hypertension, psychiatric problems, memory-related disease, and stroke. The blood samples were collected by trained nurses with 8-ml samples of fasting blood and were tested within 1–2 h by professional doctors from township hospitals or the local China Center for Disease Control and Prevention. Blood measurements included glucose, glycosylated hemoglobin, and total cholesterol. The definitions of variables are summarized in Table 1.

**Table 1:** Definitions of variables used

Baseline characteristics	Wave	Definitions
	Wave 1 (2011–2012)	
Time-invariant		
Age		Continuous
Sex		Male = 1, female = 2
BMI		Continuous
Education level		11 Categories: “Illiterate,” “Did not finish primary school but capable of reading and/or writing,” “Home school,” “Elementary school,” “Middle school,” “High school,” “Vocational school,” and four higher levels
Marital status		Living with spouse present = 1, married but not living with spouse temporarily for reasons such as work = 2, separated = 3, divorced = 4, widowed = 5, never married = 6
Smoking		Yes = 1, no = 2
Hukou status		Agricultural = 1, nonagricultural = 2
Alcohol use		Drink more than once per month = 1, drink less than once per month = 2, none of these = 3
Living status before age 16		Village = 1, city/town = 2
Health status before age 16		Excellent = 1, very good = 2, good = 3, fair = 4, poor = 5
Hypertension		Yes = 1, no = 2
Diabetes or high blood sugar		Yes = 1, no = 2
Psychiatric problems		Yes = 1, no = 2
Memory-related disease		Yes = 1, no = 2
Stroke		Yes = 1, no = 2
Glucose		Continuous
Glycosylated hemoglobin		Continuous
Total cholesterol		Continuous
Time-variant	Waves 1–3 (2011–2016)	
Cognitive performance scores		Continuous variable ranging from 0 to 21
Pulse pressure		Continuous
Systolic blood pressure		Continuous
Diastolic blood pressure		Continuous
ADL		Continuous variable ranging from 0 to 11
Depression		Continuous variable ranging from 0 to 30

*BMI* Body mass index, *Hukou* is the registration system in China created in 1955 to restrict internal population movement, especially rural-to-urban migration, divided Chinese into two categories: agricultural hukou (rural hukou) and non‐agricultural hukou (urban hukou)

### Data analysis

#### Descriptive statistics

Two-sample *t* tests and Pearson’s chi-square tests were used to examine differences in the missing data group and the nonmissing group. Spearman’s coefficients were used to test the correlations between cognitive performance and PP at three waves. One-way repeated measures analysis of variance (ANOVA) was applied to assess the changes of frequency of cognitive scores and PP in 2011, 2013, and 2015 for different age and sex groups. Then one-way ANOVA was used to compare the differences within age or sex groups.

#### Latent growth model

**Fig. 1:**
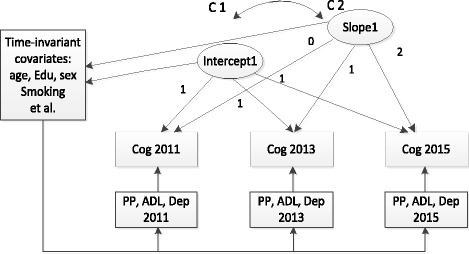
Measurement of latent growth model with time-invariant and time-variant covariates for cognitive performance. Time-invariant covariates include age, sex, education level, marital status, smoking, drinking alcohol, hypertension, stroke. *Cog* Cognitive performance, *PP* Pulse pressure, *ADL* Activities of the daily living, *Dep* Depression, *C1-C2* Correlation of intercept 1 and slope 1, *Edu* Education level

LGMs were used to examine the trajectories of changes in cognitive performance and PP. The trajectory of change in cognition across time was modeled with two latent variables: one been latent intercept growth factor, which represented the initial status of the cognition, and the other being the latent slope growth factor, which reflected the rate of change in cognition. Given the three waves of data, the growth trajectory of variable was modeled with a specified linear LGM. First, an initial LGM was applied to establish a model reflecting the change of cognitive performance over time with time-variant PP at three time points. For the intercept factor in the initial model, the loading from the factor to each of the repeated measures is set to the fixed value of 1.0. For the slope factor, we fixed the loading of the cognitive measures at the values of 0, 1.0, and 2.0. This model was compared against the conditional model, which was based on the initial model, controlled for a series of predictor variables (Fig. 1).

**Fig. 2:**
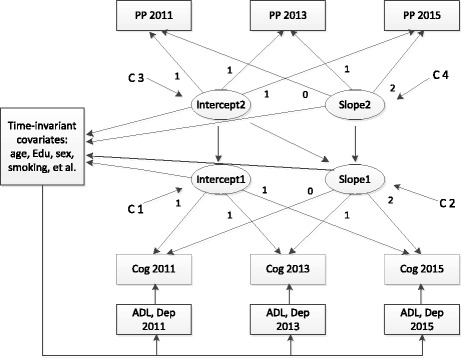
Conditional structural latent growth model to assess the relationships of the changes in pulse pressure (PP) and cognition. *Cog* Cognitive performance, *PP* Pulse pressure, *ADL* Activities of the daily living, *Dep* Depression, *C1-C2* Correlation of the intercept 1 and slope 1, *C3-C4* Correlation of the intercept 2 and slope 2, *Edu* Education level

The next step is to assess the relationships of the changes in PP with changes in cognition. Two unconditional LGMs were first modeled to reflect, respectively, the changes in the trajectories of cognitive functioning and PP with no predictor variables. The values of the intercept and slope factors were set similarly to the previous model. To test the effects of PP on the initial level and subsequent development of cognition, unconditional (including no covariates or predictors) and conditional (including covariates or predictors) models were compared sequentially. In the conditional model, the intercept and slope parameters of cognition were regressed on the intercept and slope factors of PP, the index of age, sex, smoking, alcohol use, ADL, depression, fasting glucose, glycosylated hemoglobin, total cholesterol, hypertension, stroke, and other variables served as the covariates (Fig. 2).

The following indices were used to assess the goodness of model fit: chi-square statistic, Tucker-Lewis index (TLI) ≥ 0.95, comparative fit index (CFI) ≥ 0.95, standardized root mean square residual (SRMR) ≤ 0.50, and root mean square error of approximation (RMSEA) ≤ 0.08, with 90% CI ≤ 0.08 [32, 33]. One hypothesis in this research was that data were missing at random. Statistical analyses for LGM were conducted with robust maximum likelihood estimator, a full information maximum likelihood estimation method used to estimate the parameters on the basis of all available data that provides robust estimates in the presence of nonnormality and nonindependence of observations [34–36]. Auxiliary variables related to the missing mechanism were included in the analyses to further reduce estimation bias [37].

## Results

### Descriptive analysis

After respondents without data derived from blood samples, anthropometric and physical performance, cognitive performance, and PP were excluded, the present study included 9750 participants in the final analysis. A total of 4717 respondents had some missing data in variables (except for cognition and PP) at three time points. Compared with the cases with complete data, cases with missing data were more likely to be older (59.06 years vs. 59.91 years, *P* < 0.01); to be nonsmokers (38.5% vs. 40.6%, *P* = 0.04); to drink alcoholic beverages (24.1% vs. 26.6%, *P* = 0.01); to have lower cognitive performance scores (10.47 vs. 9.83, *P* < 0.01); to have more difficulties in ADL (11.88 vs. 12.63, *P* < 0.01); to have higher SBP, DBP, and PP (129.43 vs. 132.67, *P* < 0.01; 75.30 vs. 76.49, *P* < 0.01; and 54.13 vs. 56.19, *P* < 0.01, respectively); to have agricultural hukou status (81.4% vs. 85.3%, *P* < 0.01); and to have hypertension and/or stroke (23.7% vs. 27%, *P* < 0.01; and 1.6% vs. 2.6%, *P* = 0.04, respectively). All variables were considered to be controlled in the subsequent analysis.

Table 2 presents the correlations of cognition with PP at three waves in 2011–2016. There was a negative relationship between cognition and PP. In addition, it shows that the increase in SBP and decline in DBP were associated with poor cognitive performance. To examine this negative correlation between cognition and PP in a longitudinal study, further analyses were conducted.

**Table 2:** Correlations of cognitive performance with pulse pressure in Chinese elderly persons at each time point during 2011–2016

	Cog2011	Cog2013	Cog2015	PP2011	PP2013	PP2015	DBP2011	DBP2013	DBP2015	SBP2011	SBP2013	SBP2015
Cog2011	1											
Cog2013	0.643^a^	1										
Cog2015	0.652^a^	0.705^a^	1									
PP2011	− 0.160^a^	− 0.155^a^	− 0.168^a^	1								
PP2013	− 0.136^a^	− 0.137^a^	− 0.166^a^	0.616^a^	1							
PP2015	− 0.137^a^	− 0.143^a^	− 0.160^a^	0.596^a^	0.629^a^	1						
DBP2011	0.036^a^	0.037^a^	0.027^b^	0.242^a^	0.133^a^	0.146^a^	1					
DBP2013	0.031^a^	0.030^a^	0.025^b^	0.130^a^	0.207^a^	0.128^a^	0.549^a^	1				
DBP2015	0.050^a^	0.052^a^	0.046^a^	0.115^a^	0.106^a^	0.206^a^	0.567^a^	0.556^a^	1			
SBP2011	− 0.091^a^	− 0.087^a^	− 0.101^a^	0.836^a^	0.504^a^	0.496^a^	0.735^a^	0.403^a^	0.403^a^	1		
SBP2013	− 0.080^a^	− 0.080^a^	− 0.104^a^	0.513^a^	0.833^a^	0.521^a^	0.406^a^	0.714^a^	0.392^a^	0.588^a^	1	
SBP2015	− 0.071^a^	− 0.074^a^	− 0.090^a^	0.494^a^	0.512^a^	0.837^a^	0.422^a^	0.405^a^	0.707^a^	0.583^a^	0.595^a^	1

*Abbreviations: Cog* Cognition, *DBP* Diastolic blood pressure, *PP* Pulse pressure, *SBP* Systolic blood pressure

^a^*P* < 0.01

^b^*P* < 0.05

Changes in cognition and PP for the total sample by age and sex group at three time points are summarized in Table 3. During the study period, the data showed curvilinear changes in both cognitive performance and PP in elderly participants, with a slight increase in 2013 and a subsequent decline in 2015. One-way repeated measures analysis suggested a significant change in cognition over time. Obviously, cognitive performance scores showed a steep age-related decrease in older adults between three time points (*P* < 0.01). Males exhibited better cognitive performance than their counterparts with lower cognition across time (*P* < 0.01). PP showed significant differences during the study period (*P* < 0.01). Males tended to have lower PP than females (*P* < 0.05).

**Table 3:** Levels of cognitive performance and pulse pressure in elderly Chinese participants at each time point

Variable	2011			2013			2015		
	No. of participants	Mean ± SD	*P* value^a^	No. of participants	Mean ± SD	*P* value^a^	No. of participants	Mean ± SD	*P* value^a^
Cognitive score		*F* = 127.77, *P* < 0.001^b^							
Total	9384	10.17 ± 4.34		9132	10.31 ± 4.36		9048	9.80 ± 4.46	
Age, years									
45–59	5071	10.99 ± 4.08	<0.001	5015	11.20 ± 4.07	<0.001	5068	10.73 ± 4.15	<0.001
60–64	1731	10.26 ± 4.21		1711	10.29 ± 4.15		1704	9.77 ± 4.34	
65–79	2351	8.76 ± 4.39		2248	8.67 ± 4.49		2138	7.93 ± 4.49	
80+	231	5.95 ± 3.98		158	5.82 ± 4.30		138	4.96 ± 4.04	
Sex									
Male	4349	11.19 ± 3.93	<0.001	4253	11.34 ± 3.92	<0.001	4204	10.77 ± 4.05	<0.001
Female	5030	9.29 ± 4.53		4873	9.42 ± 4.53		4839	8.97 ± 4.62	
Pulse pressure	*F* = 11.09, *P* < 0.001^b^								
Total	8853	54.98 ± 15.06		7668	55.47 ± 14.87		8171	54.51 ± 14.59	
Age years									
45–59	4563	50.38 ± 11.87	<0.001	4112	50.90 ± 11.96	<0.001	4492	50.06 ± 11.78	<0.001
60–64	1590	55.87 ± 14.42		1482	56.78 ± 14.30		1528	56.35 ± 13.92	
65–79	2185	62.29 ± 16.82		1957	62.86 ± 16.48		1992	61.88 ± 16.06	
80+	215	71.71 ± 18.38		171	69.64 ± 17.82		159	70.44 ± 20.12	
Sex									
Male	3972	54.31 ± 13.71	<0.001	3577	55.11 ± 13.92	0.045	3768	54.12 ± 14.00	0.026
Female	4577	55.56 ± 16.11		4086	55.78 ± 15.63		4397	54.84 ± 15.07	

^a^*P* value was calculated by one-way analysis of variance (ANOVA)

^b^*P* value was calculated by one-way repeated measures ANOVA

Owing to missing data for some variables, the number of cases does not always sum to 9750

### Latent growth model

Table 4 presents the estimates of the initial LGM and the adjusted model. Based on the initial model, the trajectory of the cognition and controlled PP was described by the specified linear model. The intercept of the cognition was 11.24 (*P* < 0.01), and the slope was 0.01 (*P* = 0.92). Standardized coefficients of the PP at three time points indicated negative effects on the cognitive performance in elderly Chinese participants (*P* < 0.01). However, in adjusted model, these effects were attenuated, PP at wave 1 showed no association with cognition, there were still significant negative associations at wave 2 (*P* < 0.05) and wave 3 (*P* < 0.05).

**Table 4:** Standardized coefficients for initial model and adjusted latent growth models

Models	Parameters	Standardized coefficients	Z value	*P* value	Goodness-of-fit indices
Initial model	Intercept	11.24	68.73	< 0.01	χ^2^(7) = 146.9, *P* < 0.001, CFI = 0.98, TLI = 0.97, SRMR = 0.058; RMSEA = 0.06 (0.05–0.07)
	Slope	0.01	0.11	0.92	
	PP 2011	− 0.05	− 4.80	< 0.01	
	PP 2013	− 0.04	− 5.46	< 0.01	
	PP 2015	− 0.08	− 9.20	< 0.01	
Adjusted models^a^	Intercept	8.89	6.51	< 0.01	χ^2^(33) = 128.9, *P* < 0.001, CFI = 0.99, TLI = 0.98, SRMR = 0.01; RMSEA = 0.02 (0.02–0.03)
	Slope	− 1.28	− 1.70	0.08	
	PP 2011	0.01	0.52	0.61	
	PP 2013	− 0.02	− 1.98	0.05	
	PP 2015	− 0.02	− 2.27	0.02	

*Abbreviations: CFI* Comparative fit index, *PP* Pulse pressure, *RMSEA* Root mean square error of approximation, *SRMR* Standardized root mean square residual, *TLI* Tucker-Lewis index

^a^Adjusted for age, sex, body mass index, education level, marital status, smoke, alcohol use, hukou status, living status and health status before age 16, hypertension, diabetes or high blood sugar, psychiatric problems, memory-related disease, stroke, glucose, glycosylated hemoglobin, total cholesterol, activities of daily living, and depression symptoms

The results of the measurement and structural models are summarized in Table 5. The trajectory of the PP was depicted by the linear LGM, with good fit indices (Table 5). The intercept of the PP growth trajectory showing the initial PP level was 55.13 mmHg (*P* < 0.01). In line with the results of ANOVA, the estimate of the slope was − 0.07 (*P* > 0.05), indicating a nonsignificant decline in the rate of changes in PP across three waves. The trajectory of cognition was well described with excellent goodness-of-fit indices. Both the intercept and the slope were significant, showing a typical decrease in the average rate of change in cognition during 2011–2016. In addition, the initial status of LGM was 10.48, similar to the cognitive performance (10.17) in 2011.

**Table 5:** Standardized coefficients for measurement and structural models

Models	Parameters	Coefficients	Z value	Goodness-of-fit indices
Measurement models				χ^2^(1) = 35.1, *P* < 0.001, CFI = 0.99, TLI = 0.98, SRMR = 0.01; RMSEA = 0.06 (0.04–0.08)
Trajectory of PP	Intercept	55.13^a^	357.75	
	Slope	− 0.07	− 0.91	
Trajectory of cognitive score	Intercept	10.48^a^	241.29	χ^2^(1) = 91.6, *P* < 0.001, CFI = 0.99, TLI = 0.97, SRMR = 0.02; RMSEA = 0.09 (0.08–0.10)
	Slope	− 0.28^a^	− 13.91	
Structural models				
Unconditional model	PP intercept → cog intercept	− 0.25^a^	17.28	χ^2^(8) = 125.8, *P* < 0.001, CFI = 0.99, TLI = 0.99, SRMR = 0.02; RMSEA = 0.04 (0.03–0.05)
	PP intercept → cog slope	− 0.16^a^	− 3.53	
	PP slope → cog slope	− 0.06	− 0.66	
Conditional model^b^	PP intercept → cog intercept	− 0.04^a^	− 2.56	χ^2^(68) = 285.5, *P* < 0.001, CFI = 0.99, TLI = 0.98, SRMR = 0.01; RMSEA = 0.02 (0.02–0.02)
	PP intercept → cog slope	− 0.10	− 1.34	
	PP slope → cog slope	− 0.07	− 0.63	

*Abbreviations: CFI* Comparative fit index, *Cog* Cognition, *PP* Pulse pressure, *RMSEA* Root mean square error of approximation, *SRMR* Standardized root mean square residual, *TLI* Tucker-Lewis index

^a^Statistical significance

^b^Adjusted for age, sex, body mass index, education level, marital status, smoking, alcohol use, hukou status, living status and health status before age 16, hypertension, diabetes or high blood sugar, psychiatric problems, memory-related disease, stroke, glucose, glycosylated hemoglobin, total cholesterol, activities of daily living, and depression symptoms

The unconditional structural models were used to assess the relationships of the initial status and changes in PP with initial status and changes in cognition, which were established with the satisfactory model fitness. On the basis of the results of the unconditional model, it was observed that the initial level of the PP was negatively associated with the initial level of cognition at baseline. The path standardized coefficient between two intercepts was − 0.25, which indicated the higher PP of participants and the lower cognitive performance they had initially. Similarly, the path standardized coefficient of the intercept of PP and slope of cognition (β = − 0.16) suggested that the elderly participants with higher PP had positive effects on the decline in cognitive scores compared with the participants with lower PP at baseline. Based on the path standardized coefficient of two slope growth factors, the rate of change in PP showed a nonsignificant association with the rate of change in cognitive function (β = − 0.06, *P* > 0.05).

After controlling the predictors, the conditional LGM presented better fit indices than the unconditional model. Consistent with the results of the unconditioned model, the initial status of PP was associated with cognitive scores at baseline; yet, the path standardized coefficient between them was weakened (β = − 0.04, *P* < 0.05). In contrast, the path from the intercept of PP to the slope of cognition became nonsignificant (β = − 0.10, *P* > 0.05).

Additionally, the residual variance of the intercept and slope in both PP and cognition showed significant correlations in the conditional LGM. The correlation coefficients of intercept-slope in PP and cognition were − 0.23 and 0.36, which suggests that there were strong interindividual differences in both initial status and the rate of changes for PP and cognition.

Table 6 presents the standardized coefficients for covariates in the conditional structural LGM. On the basis of the results in Table 6, it can be easily observed that participants with higher PP were prone to be older, illiterate, female, and smokers, as well as to have a higher body mass index (BMI) and hypertension at baseline. Individuals who were older, female, illiterate, living in a village before age 16, and living alone, as well as having a bad health status before age 16, higher BMI, and psychiatric problems, were more likely to have worse cognitive performance. In addition, the covariates of age, sex, education level, BMI, and health status before age 16 were identified as the relative factors of changes in cognitive performance scores.

**Table 6:** Standardized coefficients for covariates in the adjusted structural latent growth model

Covariates	PP		Cognitive score	
	Initial status	Slope	Initial status	Slope
Age	0.38^a^	0.03	− 0.11^a^	− 0.23^a^
Sex	0.04^a^	− 0.06	− 0.11^a^	0.17^a^
BMI	0.05^a^	0.02	− 0.11^a^	0.03^a^
Education level	− 0.09^a^	0.02	0.60^a^	0.27^a^
Marital status	0.07^a^	− 0.05	− 0.02^a^	− 0.05
Smoking	− 0.03^a^	0.05	0.02	0.03
Alcohol use	− 0.01^a^	− 0.04	0.01	− 0.10
Hukou status	0.00	− 0.02	0.08^a^	0.03
Living status before age 16	0.00	− 0.04	0.05^a^	− 0.05
Health status before age 16	0.01	− 0.05	− 0.05^a^	0.15^a^
Hypertension	− 0.31^a^	0.06	− 0.00	− 0.08
Diabetes or high blood sugar	0.01	− 0.06	− 0.03^a^	− 0.03^a^
Psychiatric problems	0.03^a^	0.01	0.03^a^	− 0.07
Memory-related disease	0.03^a^	− 0.02	0.01	0.09
Stroke	0.03^a^	− 0.11^a^	− 0.00	0.04
Glucose	0.07^a^	0.05	0.07^a^	0.05
Glycosylated hemoglobin	− 0.03	− 0.01	− 0.03	− 0.01
Total cholesterol	0.05^a^	− 0.01	0.05^a^	− 0.01

*BMI* Body mass index, *PP* Pulse pressure, *Hukou* is the registration system in China created in 1955 to restrict internal population movement, especially rural-to-urban migration, divided Chinese into two categories: agricultural hukou (rural hukou) and non‐agricultural hukou (urban hukou)

^a^Statistical significance

## Discussion

The results across a 5-year follow-up documented that, in a large sample of middle-aged and older Chinese adults, PP at three time points indicated negative effects on cognitive performance. These effects were attenuated as the confounders adjusted; thus, significant negative associations in PP and cognition persisted at wave 2 and wave 3. After controlling for a series of covariates, initial level of PP was negatively associated with initial level of cognitive performance. However, the association of initial status of PP and change of cognitive performance was weakened and became nonsignificant. The implication of this result demonstrates that a higher PP lowers the cognitive performance in middle-aged and elderly persons, but it is not a contributor to the rate of change in cognition.

Although the effect of PP on cognitive impairment was small on the basis of the standardized coefficients from PP to cognition, it was indeed one of the nonsociodemographic factors that contributed to cognitive impairment in our results. PP, as a modifiable risk factor, highlights the importance of public health implications that even small benefits are achievable in terms of preventing cognitive decline through maintaining or reducing PP in middle-aged and older adults, which deserves to be focused on [38]. In addition, maintaining or reducing PP to within normal limits reduces the risk of comorbid chronic diseases [39].

In agreement with our results, the findings of a study conducted by Wang et al. suggested that PP instead of BP may contribute to white matter change (WMC) progression and related cognitive impairment [24], which has also been confirmed in elderly participants in another study [40]. Yasar and colleagues [41] indicated that PP in older nondemented women increased risk for later-life cognitive impairment. Their study indicated that increased PP may confer added risk of global cognitive decline and specific impairment in language abilities, even after adjusting for the relevant vascular risk factors [42]. Another recent study demonstrated that PP was also related to greater dementia risk in midlife among participants who used antihypertensive medication [43].

Although the mechanisms linking higher PP and cognitive impairment remain unclear, there is supporting evidence for the notion of higher elevated PP as a risk factor for cognitive decline. Increased PP is a marker of increased stiffer arteries in large conduit arteries, particularly in older adults [9, 44–46]. Age-related arterial stiffening as an independent vascular risk factor has independent effects and joint effects with hypertension on cardiovascular diseases and cognitive function [47, 48], which is more likely related to the Alzheimer’s disease pathology than to other vascular risks [19, 26, 49]. When stiffening of large arteries occurs, blood vessels, especially the small vessels in the brain, are exposed to high PP and blood flow, causing cerebral small vessel disease and resulting in WMC development and progression [50, 51]. In addition, PP can potentially represent the chronic effects of hypertension, and researches have suggested that longer duration of hypertension is related to increased risk of cognitive decline and dementia [45, 46].

The strengths of this study include its prospective longitudinal design and its representative nationwide sample. CHARLS provides nationally representative panel data that enable inferences to be drawn about the Chinese population 45 years of age and older. To our knowledge, this is the first longitudinal study of PP and cognition to investigate the relationship of initial status and changes between them by using LGMs over the whole study period. Second, the present study used PP measures instead of BP itself, which can not only reflect arterial stiffness partly and but also represent the chronic effects of hypertension, which is more important in age-related cognitive decline. Third, besides assessing changes in exposure and outcome variables, in this study we also examined the effect of PP on cognition at different time points by establishing a time-variant LGM in which PP over time was viewed as a time-varying variable. Last, a comprehensive range of covariates, including sociodemographic factors, health- and lifestyle-related factors, chronic diseases, and blood samples were adjusted for to test the real association of PP and cognition.

There are also several limitations of our study. First, the attrition rate was a bit high. One of the main reasons was that quite a few participants did not have data on anthropometric and physical performance measures and blood samples. Second, the three measures in a 5-year period in our study made it impossible to scrutinize nonlinear relationships between PP and cognition; we could only simply assume them in linear association. Next, the assessment of cognition was based on only three aspects of measurement and did not assess all areas of cognitive function. In addition, the lack of molecular markers and imaging-based diagnosis for neurodegenerative diseases limited the study to examination of the mechanism linking elevated PP and cognitive impairment. Although this study revealed cognitive decline as a whole across a 5-year period of observation, suggested associations must be interpreted cautiously because they were generated at an interval that might not be long enough to discover the obvious cognitive decline. Hence, in our future research, we will continue this follow-up study and proceed with a more suitable analysis to test and verify the association of PP and cognitive function comprehensively over a longer time period.

## Conclusions

In summary, the present study suggests that the current status of PP is associated with the initial status of cognition and appears to be independent of covariates such as age, sex, ADL, depression, lifestyle, and chronic diseases across a 5-year follow-up. Further well-designed, population-based prospective studies with repeated PP measurements, as well as more detailed data over a long study period, are needed to investigate the association between the changes in PP and cognitive status.

## Additional file

Additional file 1: Final dataset. (XLSX 2360 kb)

## Abbreviations

ADL: Activities of daily living
ANOVA: Analysis of variance
BMI: Body mass index
BP: Blood pressure
CFI: Comparative fit index
CHARLS: China Health and Retirement Longitudinal Study
Cog: Cognitive performance
DBP: Diastolic blood pressure
Dep: Depression
IADL: Instrumental activities of daily living
LGM: Latent growth model
PP: Pulse pressure
RMSEA: Root mean square error of approximation
SBP: Systolic blood pressure
SRMR: Standardized root mean square residual
TICS: Telephone Interview for Cognitive Status
TLI: Tucker-Lewis index
WMC: White matter change
